# Harnessing the Potential of Halogenated Natural Product Biosynthesis by Mangrove-Derived Actinomycetes

**DOI:** 10.3390/md11103875

**Published:** 2013-10-14

**Authors:** Xue-Gong Li, Xiao-Min Tang, Jing Xiao, Guang-Hui Ma, Li Xu, Shu-Jie Xie, Min-Juan Xu, Xiang Xiao, Jun Xu

**Affiliations:** 1 College of Marine Life Sciences, Ocean University of China, Qingdao 266003, China; E-Mail: agong1983@163.com; 2 State Key Laboratory of Microbial Metabolism and School of Life Science & Biotechnology, State Key Laboratory of Ocean Engineering, Shanghai Jiao Tong University, Shanghai 200240, China; E-Mail: xoxiang@sjtu.edu.cn; 3 Key Laboratory of Marine Biogenetic Resources, The Third Institute of Oceanography, State Oceanic Administration, Xiamen 361005, China; E-Mails: frogyy@126.com (X.-M.T.); hellenx@126.com (J.X.); guanghui0527@126.com (G.-H.M.); xuli19860123@126.com (L.X.); xieshujie404@gmail.com (S.-J.X.); 4 Ministry of Education Key Laboratory of Systems Biomedicine, Shanghai Center for Systems Biomedicine, Shanghai Jiao Tong University, Shanghai 200240, China

**Keywords:** mangrove-derived actinomycetes, genome mining, halogenase, enduracidin, ansamycin

## Abstract

Mangrove-derived actinomycetes are promising sources of bioactive natural products. In this study, using homologous screening of the biosynthetic genes and anti-microorganism/tumor assaying, 163 strains of actinomycetes isolated from mangrove sediments were investigated for their potential to produce halogenated metabolites. The FADH_2_-dependent halogenase genes, identified in PCR-screening, were clustered in distinct clades in the phylogenetic analysis. The coexistence of either polyketide synthase (PKS) or nonribosomal peptide synthetase (NRPS) as the backbone synthetases in the strains harboring the halogenase indicated that these strains had the potential to produce structurally diversified antibiotics. As a validation, a new enduracidin producer, *Streptomyces atrovirens* MGR140, was identified and confirmed by gene disruption and HPLC analysis. Moreover, a putative ansamycin biosynthesis gene cluster was detected in *Streptomyces albogriseolus* MGR072. Our results highlight that combined genome mining is an efficient technique to tap promising sources of halogenated natural products synthesized by mangrove-derived actinomycetes.

## 1. Introduction

Mangroves, unique habitats in tropical and subtropical tidal areas, are known to be highly productive ecosystems [1]. There is unambiguous evidence that the mangrove ecosystem contains a large diversity of actinomycetes, which have the potential of producing anti-infection and anti-tumor bioactive secondary metabolites [2,3,4]. Structurally unique bioactive compounds have been obtained from mangrove-derived actinomycetes [4,5,6], however, how to tap the treasure trove of natural products produced by mangrove-derived actinomycetes is still of interest to drug developers.

The traditional cultivation-dependent approach of screening secondary metabolites was time-consuming and resulted in high labor costs. Additionally, intensive chemical exploitation of natural products always led to the identification of already known compounds [7,8]. These situations greatly hamper the discovery of novel secondary metabolites from microorganisms. Therefore, an effective and rational screening strategy is needed to investigate the biosynthetic potential of a large set of bacterial strains. A sequence-guided screening method is becoming more and more important for strain evaluation [9,10]. Polyketide synthase (PKS) and nonribosomal peptide synthetase (NRPS) are multi-domain enzymes or enzyme complexes that produce pharmaceutic important polyketides and nonribosomal peptides, respectively [11], but only targeting the conservative domains in either PKS or NRPS will always get more than one hit that belongs to different biosynthesis gene clusters.

The tailing steps, in general, enhance the bioactivity of the compounds, during which glycosyltransferases, methyltransferases, acyltransferases, prenyltransferases, aminotransferases, cyclases, halogenases, ketoreductases, and oxygenases, were involved to further diversify the structures of the natural products [7,12]. Thus, tailoring genes were deemed as unique indicators for hunting natural product biosynthetic genes in actinomycetes [7]. For example, using the cyclase gene that involved in the formation of aromatic ring as indicator, the angucycline-producing potential of actinobacteria was rapidly estimated by a PCR-based approach [13]. Epoxidase were proven to be a good marker for the existence of polyether biosynthetic gene clusters in the case of monensin, nanchangmycin, lasalocid, nigericin, and tetronomycin [14]. Based on polyether-specific epoxidase sequences, a degenerate primer was designed and a salinomycin biosynthesis gene cluster was cloned and characterized successfully [15].

Halogenation is an important feature for the bioactivity of a large number of distinct natural products. Chlorination was the most frequently found modification, followed by bromination, while iodination and fluorination are rare in nature [16]. FADH_2_-dependent halogenases, which introduced chloride and bromide into natural compounds, is the biggest group of specific halogenating enzymes known to date. Using FADH_2_-dependent halogenases as indicators, Gao *et al.* [17] describe an effective and rational screen strategy that can rapidly estimate the antibiotic producing potential in a large actinobacterial strain collection. They found that genes adjacent to the halogenase genes show significant identities to genes that involved in the biosynthetic gene clusters for avilamycin, viomycin, and sporolide.

To exploit the potential of halogenated natural products from mangrove-derived actinomycetes, we investigated the diversity of FADH_2_-dependent halogenases in 163 mangrove-derived actinomycetes and evaluated the potential of these strains to produce halogenated metabolites. A new enduracidin producer, *S. atrovirens* MGR140, was identified, and a putative biosynthetic gene cluster for halogenated ansamycin in *S. albogriseolus* MGR072 was proposed.

## 2. Results and Discussion

### 2.1. Potential of Biosynthesis of Halogenated Metabolites in Mangrove-Derived Actinomycetes

**Figure 1:**
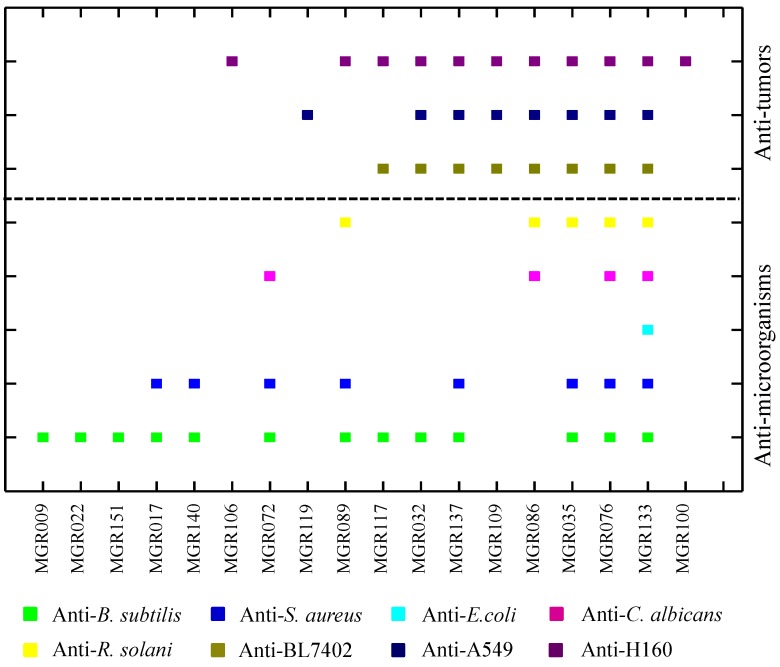
Antagonistic spectrum of halogenase positive strains. Fermentation broth was centrifuged and filtered, and use the supernatant for anti-microorganism tests or for anti-tumor tests with 50× dilution, respectively.

Mangrove forests are one of the most productive wetlands on Earth and exhibit unique biodiversity. In this study, 163 strains of mangrove-derived actinomycetes were screened for the presence of halogenase gene sequences by PCR amplification with the primers Hal3A/3B. A total of 26 different halogenase gene fragments were identified. The bioactivities of the strains harboring these halogenase genes were evaluated using anti-microorganism bioassays with five test strains and anti-tumor assays with three cell lines (Figure 1). These results indicated that the mangrove-derived actinomycetes have good potential in producing bioactive secondary metabolites.

Modular polyketide synthases (PKS-I), iterative polyketide synthases (PKS-II), and non-ribosomal peptide synthetases (NRPS) are involved in the biosynthesis of a vast array of structurally diverse natural secondary metabolites in microorganisms [18,19,20]. Gao *et al.* found that the strains containing highly homologous halogenase genes tended to produce halometabolites with similar structures [17]. Thus, the coincidence of the halogenase gene with either NRPS, PKS-I, or PKS-II genes, was checked through the specific amplification of the target genes by PCR. In the 26 halogenase-positive strains, PKS-I, PKS-II, and NRPS genes were detected (Supplementary Table S1). The strains that possessed halogenase gene and polyketide synthase genes or nonribosomal peptide synthetase genes displayed good antagonistic activities (Supplementary Table S1).

**Figure 2:**
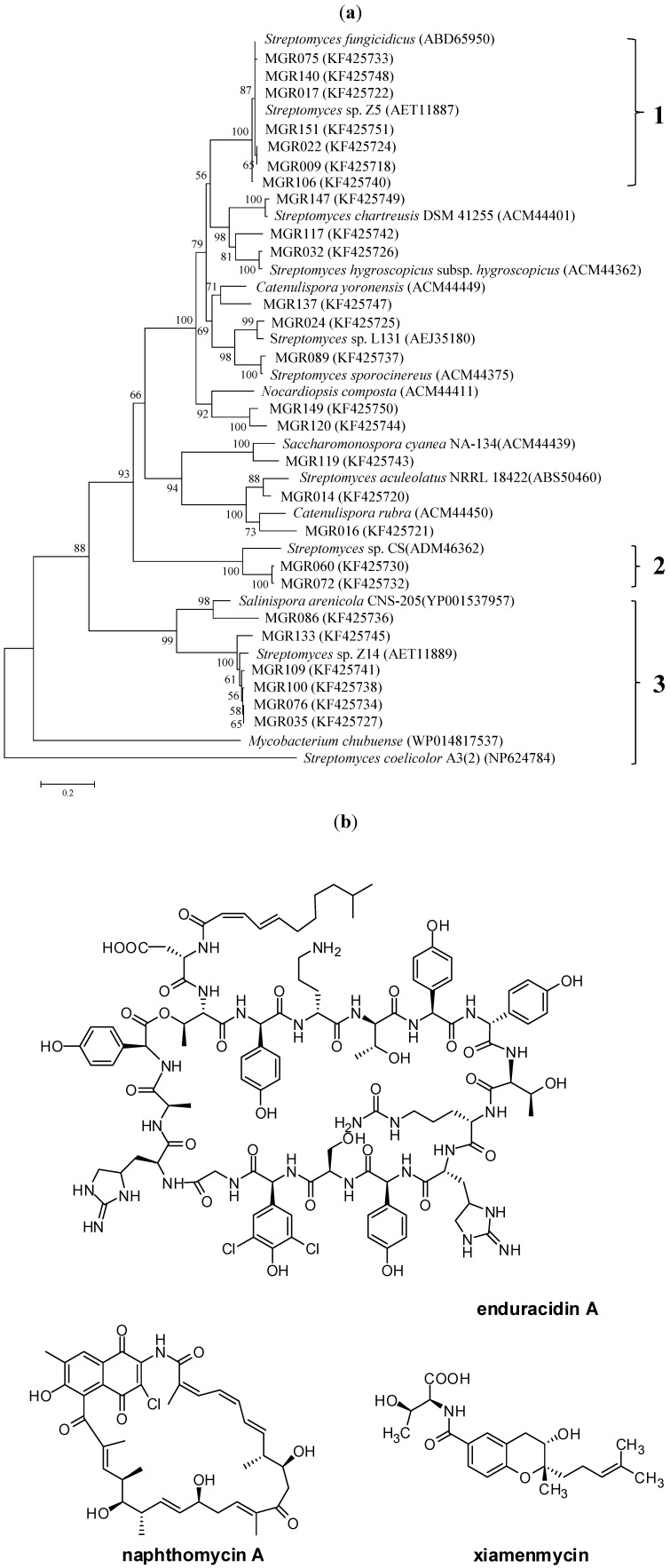
(**a**) Phylogenetic tree constructed using the halogenase sequences that were amplified with the Hal3A/3B primers. Tree topography and evolutionary distances were determined using the neighbor-joining method with 1000 replicates of bootstrapping. Bootstrap values, providing ≥50% support, are indicated. The scale bar indicates 0.2 substitutions per nucleotide position. The heme containing haloperoxidases were used as an outgroup. The numbers in the right of the close brace suggest three promising groups for genome mining; (**b**) Structures of enduracidin A [26], naphthomycin A [27], and xiamenmycin [23].

The Hal3A/3B primer pair was deduced from the conserved regions of the FADH_2_-dependent halogenases, which catalyze the chlorination of phenol- and pyrrole-containing metabolites [7]. All of the obtained sequences belonged to FADH_2_-dependent halogenases, and these sequences shared a high similarity at the amino acid level to sequences retrieved from GenBank. Phylogenetic analysis showed that these halogenase sequences were clustered into several subgroups (Figure 2a).

Group 1 is most closely related to *Streptomyces fungicidicus* (ATCC 21013), which harbors a biosynthetic gene cluster for the antibiotic enduracidin. Some strains in this clade, such as MGR140, MGR009, MGR017, and MGR151, showed a similar antimicrobial spectrum to that of enduracidin (Figure 2a). The 16S rRNA gene sequences of these strains showed closed similarity to *Streptomyces atrovirens*, suggesting that this species might be a potential producer of enduracidin.

Group 2 consisted of *Streptomyces albogriseolus* MGR072, *Streptomyces* sp. MGR060, and *Streptomyces* sp. CS. Our previous study isolated and identified a novel benzonaphthyridine alkaloid from MGR072 [21]. The clustering of MGR072 with the naphthomycin producer *Streptomyces* sp. CS and the presence of PKS-I, PKS-II, and NRPS genes in MGR072 strongly suggest that this strain possesses the potential to produce halogenated ansamycin.

Group 3 contained *Salinispora arenicola*, the representative genus of obligate marine Actinomycetales, which can produce anti-tumor compounds. Strains in group 3, except for MGR100, show broad anti-tumor bioactivity (Figure 2a). For example, *Streptomyces xiamenensis* MGR035 was identified as a novel species of mangrove-derived actinomycetes [22], and the crude extract of MGR035 exhibits versatile antagonism bioactivity including anti-bacterial, anti-tumor, anti-fibrotic, and anti-inflammatory bioactivities [23,24]. *S. xiamenensis* was first characterized as a new *Streptomyces* species that was isolated from mangrove sediment. It is interesting to find that *S. xiamenensis* has subsequently been isolated from coral and marine sponges [25], confirming that this species is widespread in the marine environment. The higher similarities in the halogenase genes of group 3 indicated that these strains may produce halogenated natural secondary metabolites due to the marine habitation features.

### 2.2. A New Enduracidin Producer *S. atrovirens* MGR140

**Figure 3:**
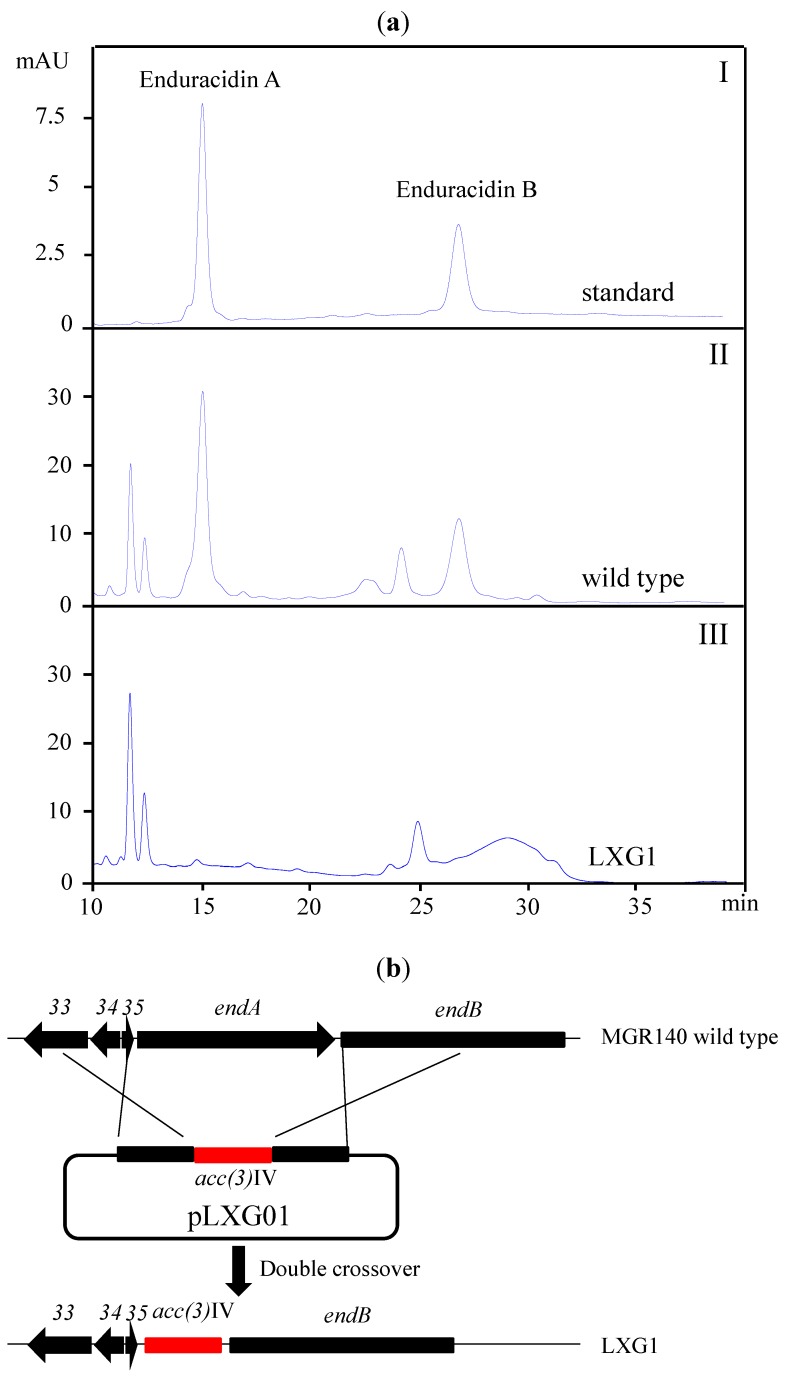
A gene cluster in *S. atrovirens* MGR140 presumably governing enduracidin biosynthesis. (**a**) HPLC analysis of enduracidin production at a UV wavelength of 267 nm. I, enduracidin standard (MP Biomedicals); II, extract from wild-type *S. atrovirens* MGR140; III, mutant LXG1 (Δ*endA*); (**b**) Construction of the *endA* mutant strain LXG1 by double-crossover gene replacement.

The halogenase gene in *S. atrovirens* MGR140 showed 100% similarity to the halogenase gene involved in enduracidin biosynthesis in *S. fungicidicus* [26]. To test whether enduracidin was produced, the fermentation broth from *S. atrovirens* MGR140 was extracted and analyzed using high performance liquid chromatography (HPLC), using standard enduracidin as a reference (Figure 3a). The fingerprints of the metabolites showed that the target compounds had the same retention time and similar UV profiles as enduracidin (Figure 3a).

The primer set P4 [28], which targeted the conserved motifs in the NRPS adenylation domains, was used to probe the enduracidin biosynthetic gene cluster in *S. atrovirens* MGR140. A total of seven clones that contained NRPS adenylation domains were found in the genomic library that consisted of 3000 fosmid clones. Further investigations were performed using the primer sets P3 and P5 for *endP* (PLP-dependent aminotransferase) and *endD* (nonribosomal peptide synthetase) amplification, respectively. Primer sets P1 and P2 were used to confirm the boundary regions of the putative enduracidin biosynthetic gene cluster. Eventually, a 116 kb region consisted of four overlapping fosmids was identified, and this region harbored the entire enduracidin biosynthetic gene cluster (Supplementary Figure S1).

To confirm the function of the cluster, the *endA* gene was replaced with the *acc(3)IV* cassette (apramycin resistance) to disrupt the putative enduracidin biosynthetic gene cluster. Regions of approximately 2 kb, flanking the *endA* and the *acc(3)IV* cassette, were cloned, resulting pLXG01 (Figure 3b). This plasmid was introduced into *S. atrovirens* MGR140 through conjugation. The crossover mutant was confirmed by PCR amplification. As expected, the mutant LXG1 strain completely lost the ability to produce enduracidin (Figure 3a). This result proved that the putative gene cluster was responsible for the enduracidin biosynthesis observed in *S. atrovirens* MGR140.

### 2.3. Identification of a Putative Halogenated Ansamycin Gene-Cluster from *S. albogriseolus* MGR072

The halogenase gene identified in *S. albogriseolus* MGR072 displayed 64% similarity to the halogenase gene involved in ansamitocin biosynthesis. Further analysis of the flanking regions of the halogenase gene in MGR072 revealed that the gene fragments encoded AHBA (3-amino-5-hydroxybenzoic acid) synthase and PKS. The high similarity between the identified genes and the rifamycin biosynthetic pathway in *Amycolatopsis mediterranei* U32 indicated that *S. albogriseolus* MGR072 may produce ansamycin antibiotics in addition to benzonaphthyridine alkaloids.

**Figure 4:**
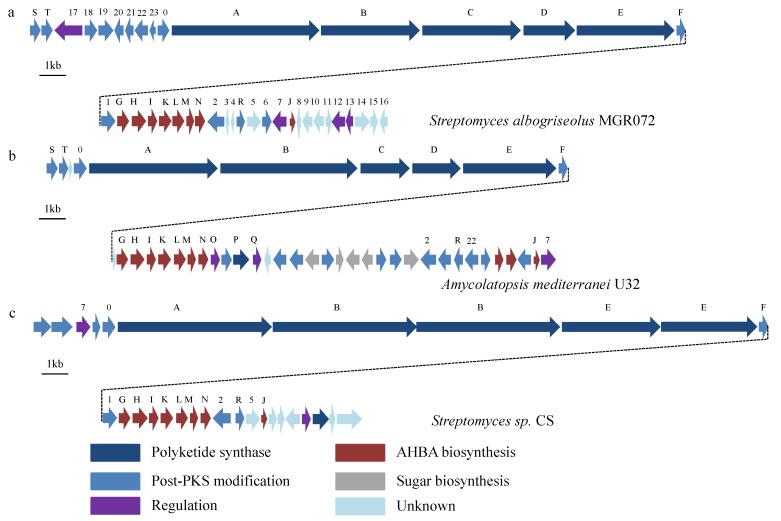
(**a**) The putative ansamycin biosynthetic gene cluster and gene organization of *S. albogriseolus* MGR072; (**b**) Rifamycin biosynthesis gene cluster from *Amycolatopsis mediterranei* U32 [29]; (**c**) Naphthomycin biosynthetic gene cluster from *Streptomyces* sp. CS [27].

As the AHBA synthase genes were highly conserved in the AHBA-derived antibiotics biosynthetic gene cluster, a special probe for AHBA synthase was used to clone the putative halogenated ansamycin biosynthetic gene cluster in *S. albogriseolus* MGR072. A total of 16 clones that contained the AHBA synthase genes were detected in the genomic library that consisted of 3000 fosmid clones. Three fosmids (5G2, 21A3, and 23G6) that contained the AHBA synthase, PKS, and halogenase were shown to reside in the putative ansamycin biosynthesis pathway. To obtain the complete putative ansamycin gene cluster, the *S. albogriseolus* MGR072 genomic library was further screened using the terminal sequence of 5G2 as a probe. Two more positive clones (26H8 and 29C9) were identified in the genomic library by sequential chromosome walking. The overlapping fosmids (Supplementary Figure S2) that covered a 84.9 kb DNA region were obtained and were tentatively named the *sha*-cluster. The predicted functions of the 41 complete open reading frames (ORFs) (ACC# KF425715) are shown in Figure 4a and Supplementary Table S2.

For the common starter unit AHBA, a conserved location and arrangement in the biosynthetic gene cluster of all ansamycin could be observed [30]. As the AHBA biosynthetic genes in rifamycin [31] and naphthomycin [27] (Figure 4b,c), the *shaGHIKLMN* genes form an operon that appears to be responsible for the biosynthesis of AHBA, while the *shaJ* gene is located 7.5 kb downstream of *shaN*. Upstream of the *sha-AHBA* cluster, a large region encoded five modular type I PKS genes (*shaA–E*) and an amide synthase gene (*shaF*), which showed similar arrangement with that in rifamycin gene cluster from *A. mediterranei* U32, but contrasted in the numbers of modulars to that in napthomycin gene cluster from *Streptomyces* sp. CS. Interestingly, *rifB* contained three KS-AT-DH-KR-ACP domains and *shaB* contained two. However, *shaC* encoding two KS-AT-DH-KR-ACP domains and *rifC* have only one. It is speculated that proteins encoded by *shaA-E* contain one loading module and ten extension modules that are capable of incorporating a total of eight propionate units and two acetate units into the AHBA starter unit. The *shaF* gene, which encodes an amide synthase, showed a high degree of similarity to the *rifF* gene of rifamycin (identity = 64%) and *natF* gene of napthomycin (identity = 57%), may catalyze the release of the completed polyketide chain from PKS, as well as generate the macrocyclic lactam.

Except the core structure biosynthetic gene cluster, the post-synthase modifications are various. Located between the *sha*-PKS cluster and *sha*-AHBA cluster is a *sha1* gene, which encoding FADH_2_-dependent halogenase, show high similarity to the *nat1* of napthomycin. It is proposed that *sha1* may relate to the halogenated modification of the proansamycin. Downstream of *shaN* is an *orf2*, that may involve in the formation of naphthalene ring. It is predicted that *orf2* catalyzes the oxidation of the tetraketide intermediate. *shaR* encoding a type II thioesterase that is predicted to removing aberrant units from carrier domains. *Orf5*, located on the downstream of *shaN*, has a sequence identity of 66% to *nat4* from naphthomycin gene cluster. *Orf7* encoding a transcriptional regulator is 44% and 48% identical to *AMED_0655* from *A. mediterranei* and *orf5* from *Streptomyces* sp. CS, respectively. Located downstream of *shaJ* is a set of genes (*orf8*, *9*, *10*, *11*, and *12*) that appear to participated in the regulation of transcription. *Orf13* encoding an EmrB/QacA family drug resistance transporter was deduced that to export putative halogenated ansamycin from *S. albogriseolus*. But functions of these genes are still unknown.

## 3. Discussion

Currently, over 4000 different natural halogen-containing compounds have been found [32]. Many of these compounds are predominately produced by microorganisms that originated in marine environments due to the relatively high halogen ion content [33]. Chloramphenicol, 7-chlorotetracyclin, and vancomycin are representative examples of antibiotics in which halogenation increases the complexity of the structure and enhances its bioactivity [34]. In addition to the enzymes involved in the natural product backbone biosynthesis, tailoring genes in the biosynthesis pathway, such as halogenases, could be used as effective probes to estimate the genetic coding potential of natural products [7,12,35,36].

There are two classes of halogenases including FADH_2_-dependent halogenases and non-heme Fe^II^-dependent halogenases that embedded in the biosynthetic gene clusters of natural products [37]. Phylogenetic analyses of FADH_2_-dependent halogenases showed that strains containing highly homologous halogenases tended to produce halometabolites with related structures [17]. To this day, almost all known FADH_2_-dependent halogenases, which form the largest class of halogenating enzymes, are involved in the halogenation of aromatic or heteroaromatic ring systems [38]. Two distinct groups of FADH_2_-dependent halogenases exist, one group that uses tryptophan as a substrate and the other that uses phenol or pyrrol [39]. Using the conserved regions of FADH_2_-dependent halogenases as probes, a sequence-guided genetic screening strategy enabled pre-selection of strains from thousands of strain collections and allowed for rapid access to the novel natural products with predetermined structural properties [7]. In addition, the non-heme Fe^II^-dependent halogenases represent a new subtype of the O_2_ and α-ketoglutarate-decarboxylating superfamily, which act on unactivated, aliphatic carbon centers [35,37]. This allows the novel halogenase as special indicator to identify the completely different classes of natural products. In this study, 26 halogenase-positive strains were screened from 163 mangrove-derived actinomycetes strains. Most of the halogenase-positive strains exhibited attractive antagonistic bioactivity, include anti-tumor, and anti-microorganism activity. Additionally, it is worth noting that there is a higher incidence of anti-microorganism bioactivity in the halogenase-positive strains compared to that of the halogenase-negative strains.

Halogenase genes might undergo widespread horizontal gene transfer (HGT) within actinomycetes [17]. Although the phylogenetic analysis of the FADH_2_-dependent halogenase genes and the 16S rRNA gene in the 26 strains showed poor consistency (Supplementary Figure S3), three promising groups of halometabolite producers could still be observed. *S. atrovirens* MGR140 in Group 1 was shown to produce the antibiotic enduracidin, whereas *S. xiamenensis* MGR035 in Group 3 was subjected to extensive studies due to its broad antagonism bioactivity [23,24]. *S. albogriseolus* MGR072 in Group 2 produced a novel benzonaphthyridine alkaloid [21], and this strain also has potential to produce halogenated ansamycin.

The putative halogenated ansamycin biosynthetic pathway in *S. albogriseolus* MGR072 has a classic core gene structure similar to that of rifamycin, though the post-PKS modifications genes are distinct. *Orf1*, which encodes a FADH_2_-dependent halogenase, is adjacent to the PKS cluster and AHBA cluster and may be involved in halogenated ansamycin biosynthesis. *Orf1* contains two typical conserved regions of this enzyme group, GxGxxG and WxWxIP (Supplementary Figure S4), and also has a flavin binding site located at the amino terminal end. *Orf1* has a sequence identity of 78% to *nat1*, which is thought to incorporate the chlorine atom at the C-30 position in naphthomycin A. The *Asm12* gene also encodes a FADH_2_-dependent halogenase and is considered to be responsible for the chlorination of ansamitocin. The well-designed cross-complementation experiment showed that the roles of *nat1* and *asm12* can be effectively taken over by each other [27]. Based on the sequence analysis, *orf1* was considered to be responsible for the chlorination of the putative halogenated ansamycin. The gene from *orf2* encodes a 3-(3-hydroxyphenyl) propionate hydroxylase, and this gene shows highly homology with the *mphA* and *nat2* genes involved in the formation of the naphthalene ring of rifamycin and naphthomycin, respectively [27,29]. *Orf2* contains three conserved regions (GXGXXG (motif I), DGXXSXXR (motif II) and GDXXH (motif III)) of the flavoprotein hydroxylases family (Supplementary Figure S5) and may catalyze the formation of the naphthalene ring of putative halogenated ansamycin. *Orf0* shows sequence similarity to cytochrome P450 hydroxylase, which is located immediately upstream of the PKS genes, and may be involved in the oxidation steps before or during the formation of the core structure of naphthoquinone. In addition, *orf22* also encodes a cytochrome P450 hydroxylase; however, this gene only has a sequence identity of 29.8% to *orf0*. The function of *orf22* could not be deduced by search and comparison with the databases. Located upstream of *orf22* are two methyltransferases that may be involved in the methylated modifications. The *shaS* and *shaT* genes encode two dehydrogenases that contain oxidoreductase domains, and these proteins are thought to catalyze naphthoquinone ring closure.

## 4. Experimental Section

### 4.1. Strain and Culture Conditions

In total, 163 actinomycetes strains were isolated from mangrove surface sediments from the Jiulong River Estuary, China. *Streptomyces* were grown at 30 °C in TSB liquid medium and SFM solid medium. *Escherichia coli* strain DH5α was used for vector construction, and propagation was cultivated in Luria-Bertani (LB) medium at 37 °C. When needed, ampicillin was added to the medium at a concentration of 100 μg/mL.

### 4.2. Detection of PKS I, PKS II, and NRPS Genes by PCR Amplifications

PCRs were run for 30 cycles. The conditions for each cycle were 1 min at 94 °C, 1 min of touchdown from 55 °C to 40 °C, and 2 min at 72 °C. Each reaction mix contained 5 ng of genomic DNA, 12.5 pmol of each primer, 1 U of Ex Taq DNA polymerase, and 10% dimethyl sulfoxide. The PCR products were purified using OMEGA Gel Extraction kits after agarose gel separation and were cloned into the pMD18-T plasmid vector (Takara Bio Inc., Tokyo, Japan) for sequencing.

The following set of PCR primers was used for the amplification the halogenase genes: Hal3A (5′-TTCCCSCGSTACCASATCGGSGAG-3′) and Hal3B (5′-GS GGGATSWMCCAGWACCASCC-3′) [7]. Another three sets of PCR primers were used: NRPS2A (5′-GCSTACSYSATSTACACSTCSGG-3′) and NRPS2B (5′-SASGTCVCCSGTSCGGTAS-3′) targeting the NRPS sequences [40]; PKS4A (5′-GCSATGGAYCCSCARCARCGSVT-3′) and PKS4B (5′-GTSCCSGTSCCRTGSSCYTCSAC-3′) targeting the type I PKS sequences [41]; PKS1A (5′-TSGCSTGCTTCGAYGCSATC-3′) and PKS1B (5′-TGGAANCCGCCGAABCCGCT-3′) targeting the type II PKS sequences [42].

### 4.3. Fosmid Library Construction and Screening

Genomic DNA from *S. albogriseolus* MGR072 and *S. atrovirens* MGR140 was prepared using the standard methods [43]. The DNA was sheared to approximately 40 kb fragments and ligated into the CopyControl pCC2FOS vector. The ligated DNA was then packaged using MaxPlax Lambda Packaging Extracts and plated on EPI300-T1R plating cells (EPICENTRE). To locate the halogenated ansamycin biosynthetic gene cluster, degenerated primers were used to screen the genomic library (AHBAF: 5′-CCSGCCTTCACCTTCATCTCCTC-3′ and AHBAR: 5′-AYCCGGAACATSGCCATGTAGTG-3′ [27]). The entire gene cluster was obtained by subcloning and sequencing the PCR products. The enduracidin cluster from *S. atrovirens* MGR140 was screened using five pairs of primers: P1 (end1F: 5′-AATGCCGACAGCCGGACAAGGT-3′/end1R: 5′-GATCCACGAAGCTCTGGTT-3′), P2 (end2F: 5′-ATCACCGCCGACAACTACGA-3′/end2R: 5′-CAGGTTCAGCATCAGCCACA-3′), P3 (end28F: 5′-TGTCAGCACATGGCGCAACGC-3′/end28R: 5′-TCATCGAGGACACGGGCAAGCT-3′), P4 (end37F: 5′-TTCACGCAGGAACGCAACAAC-3′/end37R: 5′-TGAGCGAAGGACAGCGGCAC-3′), and P5 (end40F: 5′-CTCGACAACCAGGTCAAGCT-3′/end37R: 5′-AGTTCCCGCCCAGTTCCCA-3′).

### 4.4. Gene Inactivation

Gene inactivation was carried out using standard genetic approaches. A 915 bp fragment of the *aac(3)IV* gene was amplified using the pSET152 plasmid as a template and the following primers containing *Sca*I sites: ApraR-F: 5′-AAAAGTACTTGGTTCATGTGCAGCTCCATC-3′ and ApraR-R: 5′-AAAAGTACTTGAGCTCAGCCAATCGACTG-3′. The PCR product was cloned into the pHZ1358 vector with approximately 2 kb upstream and downstream sequences of the target genes as flanking regions. The pLXG01 plasmid was constructed and introduced into *Streptomyces* sp. through conjugation with *E. coli*. The apramycin-resistant and thiostrepton-sensitive clones were selected for further verification by PCR amplification. The *endA*-deleted mutant was selected and named LXG1.

### 4.5. DNA Sequencing and Bioinformatic Analysis

DNA sequencing was carried out using Roche’s 454 sequencing platform. Approximately 237.5 Mb data, which represented 30.8-fold coverage of the genome, were produced from the Roche 454 GS FLX sequencer. Putative ORFs were predicted using the FramePlot 3.0 beta online program [44], and function annotation was performed using BLAST analysis. The PKS domains were predicted and analyzed by searching the SEARCHPKS database [45].

### 4.6. Phylogenetic Analysis

Deduced amino acid sequences of the halogenase genes retrieved from the strains were searched in the NCBI database. Related sequences were aligned using the DNAMAN program (version 5.1; Lynnon Biosoft, Quebec, Canada). A phylogenetic tree was constructed from a matrix of pairwise genetic distances using the maximum-parsimony algorithm and the neighbor-joining method in the MEGA 3.0 program, and 1000 trials of bootstrap analyses were used to provide confidence estimates for the phylogenetic tree topologies.

## 5. Conclusions

The PCR-based genetic screening approach suggests that the mangrove-derived actinomycetes harboring diversified halogenase genes are a rich source of natural products. Of the 163 mangrove-derived actinomycetes, 16% of these strains have the potential to produce various halogenated (FADH_2_-dependent halogenases) natural products. A new enduracidin producer, *S. atrovirens* MGR140, was identified. Moreover, a putative halogenated ansamycin cluster was revealed in *S. albogriseolus* MGR072. This study has applied a research strategy to screen the mangrove-derived actinomycetes for produce halogenated natural secondary metabolites.

## Supplementary Files

Supplementary File 1 Supplementary Information (PDF, 252 KB)
