# Supplementary material for: Harnessing the Potential of Halogenated Natural Product Biosynthesis by Mangrove-Derived Actinomycetes
Source: Mar Drugs. 2013 Oct 14;11(10):3875–90. doi: 10.3390/md11103875 (PMC3826140; doi:10.3390/md11103875)
Supplement: Supplementary File 1 — Supplementary Information (PDF, 252 KB) [file marinedrugs-11-03875-s001.pdf]

Supplementary Information

Table S1. Halogenase gene-positive strains detected by Hal3A/3B and information of PKS/NRPS genes and bioactivities.

Table S2. Deduced functions of ORFs in the sha-cluster from S. albogriseolus MGR072.

Figure S1. Overlapping fosmids that cover the whole enduracidin biosynthetic cluster in *S. atrovirens* MGR140.

Figure S2. Overlapping fosmids that cover the whole putative halogenated ansamycin biosynthetic cluster in *S. albogriseolus* MGR072.

Figure S3. Phylogenetic tree constructed using 16S rRNA of halogenase positive antagonistic strains.

Figure S4. Alignment of *orf1*, *nat1* and *asm12*.

Figure S5. Alignment of *orf2*, *mhpA* and *nat2*.

Table S1. Halogenase gene-positive strains detected by Hal3A/3B and information of PKS/NRPS genes and bioactivities.

Name	Accession Number	PKS I	PKS II	NRPS	Indicator Strains					Indicator Cancer Cells		
					B. subtilis	***S. aureus***	***E.coli***	***C. albicans***	***R. solani***	**BL7402**	**A549**	**H160**
MGR106	KF425740	+	+	+	−	−	−	−	−	−	−	+
MGR009	KF425718	+	+	+	+	−	−	−	−	−	−	−
MGR022	KF425724	+	+	+	+	−	−	−	−	−	−	−
MGR075	KF425733	+	+	+	−	−	−	−	−	−	−	−
MGR017	KF425722	+	+	+	+	+	−	−	−	−	−	−
MGR151	KF425751	+	+	+	+	−	−	−	−	−	−	−
MGR140	KF425748	+	+	+	+	+	−	−	−	−	−	−
MGR060	KF425730	+	+	+	−	−	−	−	−	−	−	−
MGR072	KF425732	+	+	+	+	+	−	+	−	−	−	−
MGR119	KF425743	+	+	+	−	−	−	−	−	−	+	−
MGR016	KF425721	+	+	+	−	−	−	−	−	−	−	−
MGR014	KF425720	+	+	+	−	−	−	−	−	−	−	−
MGR117	KF425742	−	+	+	+	−	−	−	−	+	−	+
MGR032	KF425726	+	+	+	+	−	−	−	−	+	+	+
MGR147	KF425749	+	+	+	−	−	−	−	−	−	−	−
MGR024	KF425725	+	−	+	−	−	−	−	−	−	−	−
MGR089	KF425737	+	−	+	+	+	−	−	+	−	−	+
MGR149	KF425750	−	+	+	−	−	−	−	−	−	−	−
MGR120	KF425744	+	+	+	−	−	−	−	−	−	−	−
MGR137	KF425747	+	+	+	+	+	−	−	−	+	+	+
MGR133	KF425745	−	+	+	+	+	+	+	+	+	+	+
MGR035	KF425727	+	+	+	+	+	−	−	+	+	+	+
MGR076	KF425734	+	+	+	+	+	−	+	+	+	+	+
MGR100	KF425738	−	−	+	−	−	−	−	−	−	−	+
MGR109	KF425741	−	−	+	−	−	−	−	−	+	+	+
MGR086	KF425736	−	+	+	−	−	−	+	+	+	+	+

Table S2. Deduced functions of ORFs in the *sha*-cluster from *S. albogriseolus* MGR072.

Protein	Amino Acids	Protein Homolog	positive/ Identify	GenBank Accession No.	Proposed Function
ShaS	315	*RifS, A. mediterranei* U32	80/70	YP003762835	dehydrogenase
ShaT	338	*RifT, A. mediterranei* U32	50/39	YP003762836	dehydrogenase
Orf17	964	*Strvi_4056, S. violaceusniger* Tu 4113	54/42	YP004813994	transcriptional regulator
Orf18	391	*Strvi_8510, S. violaceusniger* Tu 4113	61/48	YP004818099	aromatic prenyltransferase
Orf19	475	*Strvi_8511, S. violaceusniger* Tu 4113	82/72	YP004818100	tryptophanase
Orf20	274	*Mar181_2538, M. posidonica*	51/32	YP004482488	type 11 methyltransferase
Orf21	239	*AMED_2499, A. mediterranei* U32	71/56	YP003764696	transferase
Orf22	418	*AMED_0649, A. mediterranei* U32	70/55	YP003762871	cytochrome P450
Orf23	152	*acpD, A. mediterranei* U32	60/39	YP003768008	NADH-azoreductase
Orf0	363	*AMED_0616, A. mediterranei* U32	84/76	YP003762838	cytochrome P450 hydroxylase
ShaA	5,464	*RifA, A. mediterranei* U32	78/71	YP003762839	PKS domains
loading					KR-ACP
Module1					KS-AT-DH-KR-ACP
Module2					KS-AT-ACP
Module3					KS-AT-KR-ACP
ShaB	3,626	*RifB, A. mediterranei* U32	79/72	YP003762840	PKS domains
Module4					KS-AT-DH-KR-ACP
Module5					KS-AT-DH-KR-ACP
ShaC	3,614	*RifC, A. mediterranei* U32	75/66	YP003762841	PKS domains
Module6					KS-AT-DH-KR-ACP
Module7					KS-AT-DH-KR-ACP-ACP
ShaD	1,854	*RifD, A. mediterranei* U32	77/68	YP003762842	PKS domains
Module8					KS-AT-DH-KR-ACP
ShaE	3,591	*RifE, A. mediterranei* U32	78/70	YP003762843	PKS domains
Module9					KS-AT-DH-KR-ACP
Module10					KS-AT-DH-KR-ACP
ShaF	259	*RifF, A. mediterranei* U32	75/64	YP003762844	N-acetyltransferase/amide synthase
Orf1	447	*Nat1, Streptomyces sp.* CS	88/78	ADM46362	halogenase
ShaG	356	*RifG, A. mediterranei* U32	84/78	YP003762846	aminodehydroquinate synthase
ShaH	445	*RifH A. mediterranei* U32	72/64	YP003762847	aminoDAHP synthase
ShaI	270	*RifI, A. mediterranei* U32	77/70	YP003762848	aminoquinate dehydrogenase
ShaK	386	*RifK, A. mediterranei* U32	87/78	YP003762849	AHBA synthase
ShaL	362	*RifL, A. mediterranei* U32	76/69	YP003762850	Oxidoreductase
ShaM	209	*RifM, A. mediterranei* U32	87/80	YP003762851	phosphoglycolate phosphatase
ShaN	300	*RifN, A. mediterranei* U32	75/65	YP003762852	kanosamine kinase
Orf2	553	*mhpA, A. mediterranei* U32	75/68	YP003762868	FAD-dependent oxidoreductase
Orf3	64	Fer, *A. mediterranei* U32	72/61	YP003763095	Ferredoxin
Orf4	54	*ST1928_p132, S. rochei*	80/73	NP-851508	probable thioesterase
ShaR	244	*RifR, A. mediterranei* U32	81/66	YP003762870	Thioesterase
Orf5	445	*Nat4, Streptomyces sp.* CS	79/66	ADM46372	membrane ion antiporter
Orf6	280	*SACTE_0864, Streptomyces sp. SirexAA-E*	87/81	YP004801333	metallophosphoesterase
Orf7	438	*AMED_0655, A. mediterranei* U32	55/44	YP003762877	transcriptional regulator
ShaJ	144	*RifJ, A. mediterranei* U32	92/84	YP003762876	aminoDHQ dehydratase

Table S2. *Cont.*

Orf8	80	*SclaA2, S. clavuligerus ATCC 27064*	85/64	ZP08220974	DNA primase/helicase
Orf9	305	*Strvi_6475, S. violaceusniger* Tu 4113	93/91	YP004816175	resolvase domain-containing protein
Orf10	166	*Terracoccus sp. 273MFTsu3.1*	72/62	WP020141764	hypothetical protein
Orf11	417	*H340_20018, S. mobaraensis* NBRC 13819	85/79	ZP23080866	oxidoreductase
Orf12	198	*SPW_4890, Streptomyces sp.* W007	92/84	WP007454088	putative transcriptional regulator
Orf13	487	*Francci3_2757, Frankia sp.* CcI3	70/58	YP481846	EmrB/QacA family drug resistance transporter
Orf14	210	*SPW_4891, Streptomyces sp.* W007	84/71	WP007454090	putative methyltransferase
Orf15	221	*SteO22.29c, S. tenjimariensis*	90/85	CAI59998	putative transposase
Orf16	204	*SteO22.29c, S. tenjimariensis*	86/83	CAI59998	putative transposase

Figure S1. Overlapping fosmids that cover the whole enduracidin biosynthetic cluster in *S. atrovirens* MGR140. The positions of the five sets of primers (P1–P5) used to located the enduracidin biosynthetic cluster from *S. atrovirens* MGR140 are labeled.

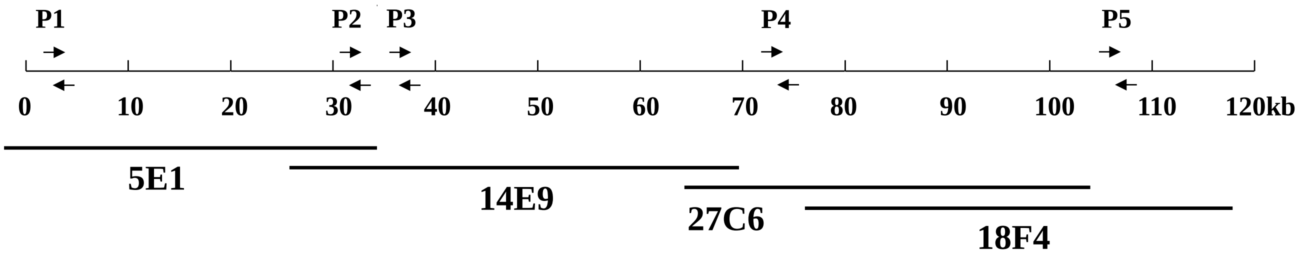

Figure S2. Overlapping fosmids that cover the whole putative halogenated ansamycin biosynthetic cluster in *S. albogriseolus* MGR072.

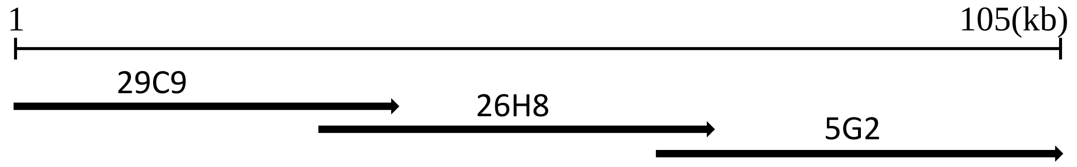

Figure S3. Phylogenetic tree constructed using 16S rRNA of halogenase positive antagonistic strains. Tree topography and evolutionary distance are given by a neighbor-joining method with 1,000 replicates of bootstrapping. Bootstrap values providing ≥ 50% support are indicated. The scale bar indicates 0.01 substitutions per nucleotide position. The dashed rectangle indicated the clade of putative enduracidin producer.

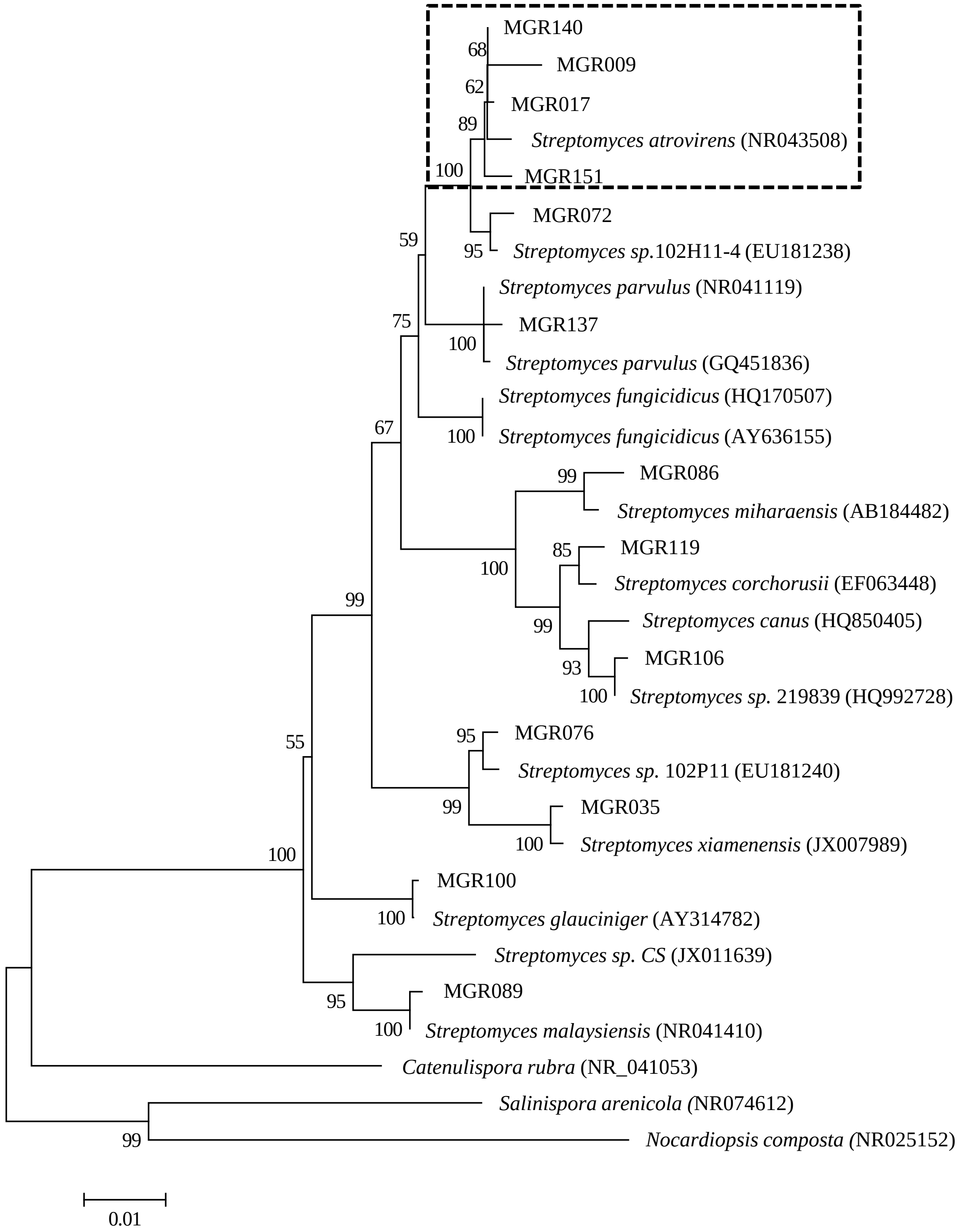

Figure S4. Alignment of *orf1*, *nat1* and *asm12*. *Orf1*, halogenase of shamycin biosynthesis from *S. albogriseolus* MGR072; *nat1*, halogenase of naphthomycin biosynthesis from *Streptomyces* sp. CS; *asm12*, halogenase of ansamitocin biosynthesis from *Actinosynnema pretiosum* ATCC31565; motif I, GxGxxG; motif II, WxWxIP.

```
                   Motif I
orf1    MAKETADVVVIGGGPAGSVCAYVLAKQGHSVVLLEKELSSRFHIGESLLPYMMGLFERIG     60
nat1    MSDNSTDVVVIGGGPAGALSAYQLAKQGHSVVLLEREEFPRFHIGESLLPYMMGLFDRIG     60
asm12   ....MLDAIVMGGGPAGSVCAAVLARQGRSVLVLERQEFPRFHIGESMLPYMVGLLERHG     56
Consensus     d  v gggpag   a  la qg sv  le     rfhiges lpym gl  r g

orf1    LRETVAAQGYVPKFGGEFIDPTEKKFFEG.VFRADFTKQGVGRHDNAFQVERAKFDRMLA    119
nat1    IREAVETKGYVPKFGGEFIDPNEEKFFSD.VFRADFSKQGDGRYDRAFQVERAKFDGMLV    119
asm12   LLDAVREQGYVVKRGGEFIDPTGTKFFRAGVFRADFAKTGDGRHHETFQVERSHFDRVNL    116
Consensus   v   gyv k ggefidp   kff   vfradf k g gr    fqver  fd

orf1    EQAEAAGARVLFGANVNELLMDGDRMVGVRYERDGESHEVSSAYVVDASGRSGRIANRFG    179
nat1    EEAGKAGAEVHLGATVGELLMEGDRMVGVRYTKDGQEHEVRSRYVVDASGRAGRVAHKFG    179
asm12   DQARAAGATVREGAQVVGLLEEGGRVVGVRYREGGVEREERARYVVDATGRAGVVANRFG    176
Consensus a  aga v  ga v  ll  g r vgvry   g   e    yvvda gr g  a  fg
                                                Motif II
orf1    LRKTLEKLRMVAVFRHYSGLDERHNPGVEGDIQVGAHDDGWVWAIPLTKDTISVGTVMPR    239
nat1    LRERLEKLRMVAVFRHYEGLDEKHNLGVEGDIQVGAHADGWVWAIPLSKDVISVGTVMPR    239
asm12   LRRMIEDLRMVAVFHHRDGLDEAHNPGHEGDIQVGSHSDGWIWAIPLSADRISVGTVMHR    236
Consensulr   e lrmvavf h  glde hn g egdiqvg h dgw waipl  d isvgtvm r

orf1    DVLRGATTQERFDEHLARVPRIVARLTGTRPSMDLKVETDYCYHTDTVTGPGWLMVGDAG    299
nat1    DVFRASTPEEVFEEHKARIPRIVARLTDTRPTMDLKVETDYCYHSDTVTGPGWVMVGDAG    299
asm12   DRLRGRTPAEAFAEHVERVPRINQRLTGTSATSDFWVETDYSYHSDQVTGPGWVMVGDAG    296
Consensud  r  t  e f eh  r pri  rlt t    d  vetdy yh d vtgpgw mvgdag

orf1    CFGDPMFSGGVLVATATAVRAAETLGEVLADPSAEDRLLERYSNYFKTGYDTYIRLIHSY    359
nat1    CFGDPMFSGGVLVASATAVRAAETLSDALNNPEDADRLIDEYANFFKTGYDTYIRLIHAF    359
asm12   CFGDPMFSGGVLVGMATGAEAAEALGRALDSPADEEQALTGYSNFFKTGYDTYVRLIFSF    356
Consensucfgdpmfsggvlv  at   aae l   l  p         y n fktgydty rli

orf1    YDGELVAMAADAARST...DRETLEKYLMRLIGGDFWSEHNSVAQEMRRRKEWDTFEPFQ    416
nat1    YDGELVAVAADAGRTT...DRETLERYLIRLIGGDFWSEHNAVAQEMRRRTEWDTFSPFQ    416
asm12   YEGELLPALAEAHSEAGNLSEADMEMYVVRLLGGDFWSARNPVANALRANPAWSTFSPFE    416
Consensuy gel    a a            e y  rl ggdfws  n va   r    w tf pf

orf1    RVYGCPSYPHLDELDRKERSEARVRRVTAG                                  446
nat1    RVFGCPSYPHLDEQDRKERAESRVRRVTQG                                  446
asm12   PVHRCPVYPELDVAELGGLAGTVGR.....                                  441
Consensusv  cp yp ld            r
```

Figure S5. Alignment of *orf2*, *mhpA* and *nat2*. *orf2*, oxidoreductase of shamycin biosynthesis from *S. albogriseolus* MGR072; *mhpA*, oxidoreductase of rifamycin biosynthesis from *A. mediterranei* U32; *nat2*, oxidoreductase of naphthomycin biosynthesis from *Streptomyces* sp. CS; motif I, GXGXXG; motif II, DGXXSXXR; motif III, GDXXH.

```
                                                        Motif I
orf2   .........MSTTRPHDP.........YESPTDTGTDT.....DVAVVGNGPVGAALSVL      37
mphA   ..................................MTDT.....DVVIVGNGPIGATLSVL      21
nat2   MSPGRTLTRSHPTRAREPGRTEGVSMSVVSPDDAMTSTSSPEADVLVVGYGPIGSALAVL      60
Consensus                                 t t     dv  vg gp g  l vl

orf2   LAQRGLRVTVLERRPRPYLLPRATSFDGETARLLAATGVGDRFGEITEPATGYQWHTAAG      97
mphA   LAQRGWRVTVLERRPRPYKLPRATSFDGETARLLAATGIGPDLGRITAPANGYQWQTADG      81
nat2   LARRGWRVTVLERRRRPYTLPRATSFDGETARLLAGTGVGGELGRITEPGTGYQWRTADG     120
Consensla rg rvtvlerr rpy lpratsfdgetarlla tg g   g it p  gyqw ta g

orf2   ETLLDIAFSPTGRYGWPDANTMHQPALERLLADRAAALPGVTVLRGRRVVDLAERDDRVS     157
mphA   QTLLDIAFTTDGPYGWPDANTMHQPALEELLAARAADLPGITVLRGHEVVAITDGDSFVQ     141
nat2   KTLLDIEFTTEGPYGWPDANTMHQPALEELLAARAGELRDITVVRDRRVVDIADGPAGVT     180
Consensutlldi f   g ygwpdantmhqpale lla ra  l   tv r   vv        v
                          Motif II
orf2   LTVETDDGAARTVVAHWVVGCDGANSFVREHLGVPMTDLGFSYEWLLCDVELREPREFTP     217
mphA   VTATGEDDVPRTISGRWVVGCDGANSFVREHLDVPVTDLGFSYEWLLCDVRLNEPREFVP     201
nat2   VTAEDDEGATRTFSARWVVGCDGANSFVRDRMGVSVTDLGFSYEWLLCDVELREPRAFVP     240
Consensut        rt    wvvgcdgansfvr    v  tdlgfsyewllcdv l epr f p

orf2   TNVQICDPARPTTLVGSGPGHRRWEFMRLPGESSAELNRPETAWRLLKPFGVTPETATLL     277
mphA   TNVQICDPARPTTLVGSGPGRRRWEFMRLPGENAAELNKDETAWRLMAPFGVTPETATLL     261
nat2   TNVQLCDPVRPTTLVGSGPGHRRWEFMRLPGERAADLNREETAWRLLAPFGVTPDTARLL     300
Consenstnvq cdp rpttlvgsgpg rrwefmrlpge  a ln  etawrl  pfgvtp ta ll
                           Motif III
orf2   RSTTYIFRAAWADEWRSGRVLLAGDAAHLMPPFAGQGMCSGIRDAANLAWKLDLTLRGLA     337
mphA   RSTTYIFQARWADRWRVGHVLLAGDAAHLMPPFAGQGMCSGIRDVTNLAWKLDLTLRGLA     321
nat2   RSTTYIFQARWADEWRLGHMLLAGDAAHLMPPFAGQGMCSGVRDVANLAWRLDLVLRGTA     360
Consensrsttyif a wad wr g  llagdaahlmppfagqgmcsg rd  nlaw ldl lrg a

orf2   DASLLDTYAEERRQHVKESILSSVQLGRVICVTDPAAAAERDATVLAGRRGRTGPGAPDA     397
mphA   PESLLDSYGDERREQAREAILASVQLGRVICVTDPAAAAERDSTVLANRRGKP.AGRPEP     380
nat2   PESLLDTYTEERKAQVRESILASVQLGRLICVTDPAAAAERDATVLANRRGTG...RPEP     417
Consensusslld y  er     e il svqlgr icvtdpaaaaerd tvla rrg      p

orf2   AKPLSAGLLRAPAGAPRA....PAGEVVPQWRVSTAGGTGLFDAVVGGGFVLLTTDDPRP     453
mphA   AKPLTGGLRHESPG.........AGVVVPQGRVQVGDATGLFDDVIGRGFVLLTTEETHS     431
nat2   ARPITCGLLHRPEGAAADTRVPPAGEVLPHARLLRPSGGEPLEDPVGRGFVLFTASEAEP     477
Consensasp   gl     g         ag v p  r              g gfvl t

orf2   ALGEDRWSFLAALDTRVVRLSPPGTVLPPEDTEP.........GLTDVIDTDGFYRAYLS     504
mphA   .......DFLTELGAHVVRLDEG.........................VDVDDVYRPFLA     459
nat2   DLTPERLSFLREVGARVVRLWPDGTSHGATDGTDGTDGTDGTDGLVDAVDADGVLRGFLG     537
Consensus      fl      vvrl                             d d   r  l

orf2   EHDASALMVRPDHHVFGAARGAEDTAALVDCLRDRLATAVPAGGPHRR                 552
mphA   RFGAASVLVRPDYHVFGTA.GPGGLEALVGALRDRLRAAVPTG.....                 501
nat2   RYGATALLVRPDYHVFGAAADPAGVAALVDDLRTRLTAPVPAPAGRAD                 585
Consensus a    vrpd hvfg a       alv  lr rl   vp
```
